# Adaptations to cognitive behavioral therapy for insomnia to address fear of sleep and its sequential impact on posttraumatic stress disorder following cognitive processing therapy

**DOI:** 10.1093/sleepadvances/zpaf078

**Published:** 2025-10-31

**Authors:** Wilfred R Pigeon, Westley A Youngren, Hugh F Crean, Victoria West Staples, Todd M Bishop, Catherine Cerulli, Autumn M Gallegos, Kathi L Heffner

**Affiliations:** Department of Psychiatry, University of Rochester Medical Center, Rochester, NY, United States; Department of Psychology, University of Missouri–Kansas City, Kansas City, MO, United States; School of Nursing, University of Rochester Medical Center, Rochester, NY, United States; Department of Veterans Affairs, Finger Lakes Healthcare System, Canandaigua, NY, United States; Department of Psychology, University of Missouri–Kansas City, Kansas City, MO, United States; Department of Psychiatry, University of Rochester Medical Center, Rochester, NY, United States; Department of Veterans Affairs, Finger Lakes Healthcare System, Canandaigua, NY, United States; Department of Psychology, University of Rochester, Rochester, NY, United States; Department of Psychiatry, University of Rochester Medical Center, Rochester, NY, United States; School of Nursing, University of Rochester Medical Center, Rochester, NY, United States; Department of Medicine, University of Rochester Medical Center, Rochester, NY, United States

**Keywords:** cognitive behavioral therapy, insomnia-comorbid, posttraumatic stress disorder

## Introduction

Posttraumatic stress disorder (PTSD) is associated with a range of comorbidities, including cardiovascular health, immune function, depression, suicide, and sleep disturbance [1–3]. Sleep disturbances are considered a “hallmark” symptom of PTSD [4, 5], with especially insomnia often persisting after PTSD pharmacotherapy and trauma-focused psychotherapies [6]. Theoretical work and empirical work support a complex relationship between sleep disturbance and the development of PTSD, with posttraumatic nightmares and trauma-induced insomnia proposed to have distinct pathologic pathways [7, 8].

Werner and colleagues [9] proposed that trauma-induced insomnia may develop partially via fear of sleep (FoS), which they define as comprising “(1) the emotional experience of fear in relation to sleep; (2) dysfunctional beliefs about safety during sleep, loss of control, and the experience of nightmares; and (3) maladaptive behavior related to fear and these beliefs.” Although FoS has not been linked to any specific type of trauma and FoS may occur in the absence of PTSD or trauma exposure, it is highly prevalent in trauma samples and well established that FoS is positively associated with the severity of insomnia, PTSD, and nightmares [10–15]. Accordingly, FoS can be considered a clinical construct and legitimate treatment target for trauma-related sleep disturbances [16]. Werner and colleagues highlighted the need to assess how trauma and sleep-focused treatments affect FoS and whether treating FoS could impact sleep symptoms related to trauma. The only study to do so is a secondary analysis of a randomized clinical trial (RCT) comparing cognitive behavioral therapy for insomnia (CBTi) to waitlist control in a mixed sample (*N* = 45) with two decades of chronic PTSD and already receiving PTSD treatment [15]. In this trial, as measured by the Fear of Sleep Inventory (FoSI) [17] a greater reduction in FoS was observed in the CBTi condition compared to the control condition posttreatment. Although the sample was relatively small, these seminal results suggest CBTi can reduce FoS severity. CBTi was not adapted to address FoS, raising the possibility that specifically addressing FoS in the context of CBTi may further enhance effects.

We address this possibility in a study wherein CBTi was adapted to address FoS. We do so in a sample with relatively more recent trauma exposure and more limited experience with PTSD treatment. The current study’s primary aim was to determine whether and to what extent FoS decreases after this adapted CBTi intervention. A second aim was to examine whether changes in FoS following CBTi augment any reductions in PTSD severity following the sequential delivery of cognitive processing therapy (CPT) for PTSD.

## Methods

We conducted a secondary analysis of a registered RCT (NCT01743339), which is further detailed elsewhere [18, 19]. The parent study randomized 110 participants with PTSD and insomnia to the sequential delivery of 4-session CBTi and 12-session CPT (CBTi + CPT; *n* = 56) or 4-session attention control followed by CPT (control + CPT; *n* = 54).

### Participants

Participants aged 18–64 years were recruited from a county domestic violence family court, a local domestic violence survivor emergency shelter, and through community referrals. The inclusion criteria included a past year index event related to interpersonal violence (IPV) resulting in moderate or severe PTSD symptoms of *>*51 on the PTSD Symptom Checklist - Specific [20] and meeting the 1-month duration criterion; full or subthreshold PTSD, with the latter defined as (1) exposure to a traumatic event, (2) at least one re-experienced symptom, and (3) either three avoidance or two arousal symptoms from the DSM-IV diagnostic criteria [21]; moderate depression of >10 on the Patient Health Questionnaire [22]; clinically meaningful insomnia of ≥10 on the Insomnia Severity Index [23]; and meeting research diagnostic criteria for insomnia disorder [24].

The exclusion criteria included pregnancy; cohabiting with their IPV perpetrator; evidence of dementia or cognitive impairment; history of schizophrenia or bipolar I disorder; current suicidality with either a plan, intent, or attempt in the past 6 months; health conditions with immunological components or undergoing immunosuppressive therapies; active alcohol dependence or remission <3 months; use of antipsychotics, opiate analgesics, and/or sleep medication; and an Apnea–Hypopnea Index *>*10 or a periodic limb movement index with arousals *>*10 assessed by in-laboratory overnight polysomnography.

### Randomization and masking

Randomization occurred through Wei urn model randomization [25] and included two strata: gender and recruitment site. The principal investigator was not blinded and communicated assignment to the CBTi or attention control therapist. Study recruiters, assessors, CPT therapists, and research assistants entering data were blind to study condition.

### Interventions

#### CBTi

Four sessions were delivered in person. Although CBTi typically comprises six to eight sessions, comparable effects on insomnia improvement have been demonstrated when standardized intervention components are delivered over four sessions [26, 27]. Participants’ responses on their baseline FoSI identified thoughts and behaviors to address within CBTi components that were delivered as follows: stimulus control therapy including establishment of a prebedtime routine (session 1), sleep psychoeducation (with an additional emphasis on FoS education; sessions 1 and 2), sleep restriction therapy (session 2), sleep hygiene (session 2), cognitive therapy (with added emphasis on reframing and processing thoughts related to FoS; session 3), and self-management/relapse prevention (session 4).

#### Control

Control participants received four weekly phone calls that included brief check-ins, safety assessments and protocol reminders. All the participants (CBTi and control) completed daily sleep diaries during this phase.

#### Cognitive processing therapy

Following the CBTi or control period, all the participants were provided with in-person CPT, the trauma-focused psychotherapy consisting of a standard, structured, 12-session, weekly protocol [28]. Each intervention is more fully described in the main findings from the parent study [18].

### Measures and assessment points

Assessments of FoS and PTSD were conducted: (1) prior to randomization; (2) approximately 1 week following CBTi or attention control; and (3) following CPT. FoS was measured with the 23-item FoSI [17], which assesses distress, fear, and anxiety related to falling and staying asleep with 5-point (0–4) Likert-style items. Items are summed for a total score where higher values indicate greater fear of going to sleep. The FoSI has demonstrated acceptable internal reliability and been validated in different samples [12, 29–31]. The gold-standard Clinician Administered PTSD Scale (CAPS) was used to assess PTSD [32].

### Statistical analysis

Unstandardized path analytic models [33–35] were used to examine aim 1 and aim 2 via the Mplus statistical software package [36]. All the variables are observed variables, with the CAPS total severity score representing PTSD symptomatology and the FoSI total score representing FoS. To account for missing data, 200 complete datasets from the original trial [18] were imputed in accordance with research supporting that more imputations are generally better within experimental conditions [37–40]. To examine the robustness of findings, sensitivity analyses were used to view results under a range of different but plausible outcomes with estimates following equations presented in Imai and colleagues [41].

## Results

Our sample was predominantly female (97%), with an average age of 35.5 years (SD = 10.9). Annual income “under $20 000” was reported by 65 per cent of the sample, 56 per cent were from a racial or ethnic minority, 27 per cent were using antidepressant medication, and 88 per cent had obtained a court order of protection upon study entry. There were no statistical differences across conditions for these and other demographic variables [18]. However, higher levels of depression severity and PTSD severity were observed in the CBTi + CPT condition, which were each statistically controlled for in analyses.

**Figure 1:**
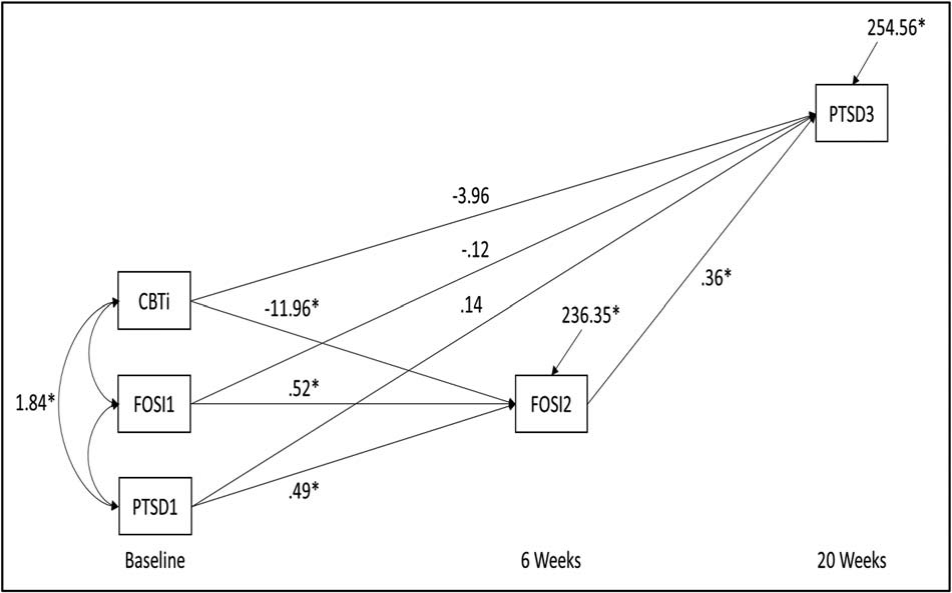
Path analysis results of CBTi effects on fear of sleep and subsequent PTSD symptoms. CBTi, cognitive behavioral therapy for insomnia; FoSI, Fear of Sleep Inventory score; PTSD, posttraumatic stress disorder severity. Straight arrows represent hypothesized causal connections (regression parameters) and double-headed curved arrows represent correlations. Gender, age, minority status, education, recruitment site, antidepressant medication class, number of traumatic lifetime events, and baseline depression covariates omitted for visual clarity.

Results of the path analysis are graphically depicted in Figure 1. Regarding aim 1, the results demonstrate that CBTi significantly decreased FoS scores at 6 weeks (*b* = −11.96, *p* = .002, 95% CI = −19.90 to −4.61). Regarding aim 2, analyses revealed that CBTi’s direct effect on PTSD was not significant (*b* = −3.96, *p* = .334, 95% credibility interval [CI] = −12.01 to 4.08) but was mediated via CBTi’s effects on FoS (*b* = −4.16, *p* = .008, 95% CI = −9.26 to −0.82).

**Figure 2:**
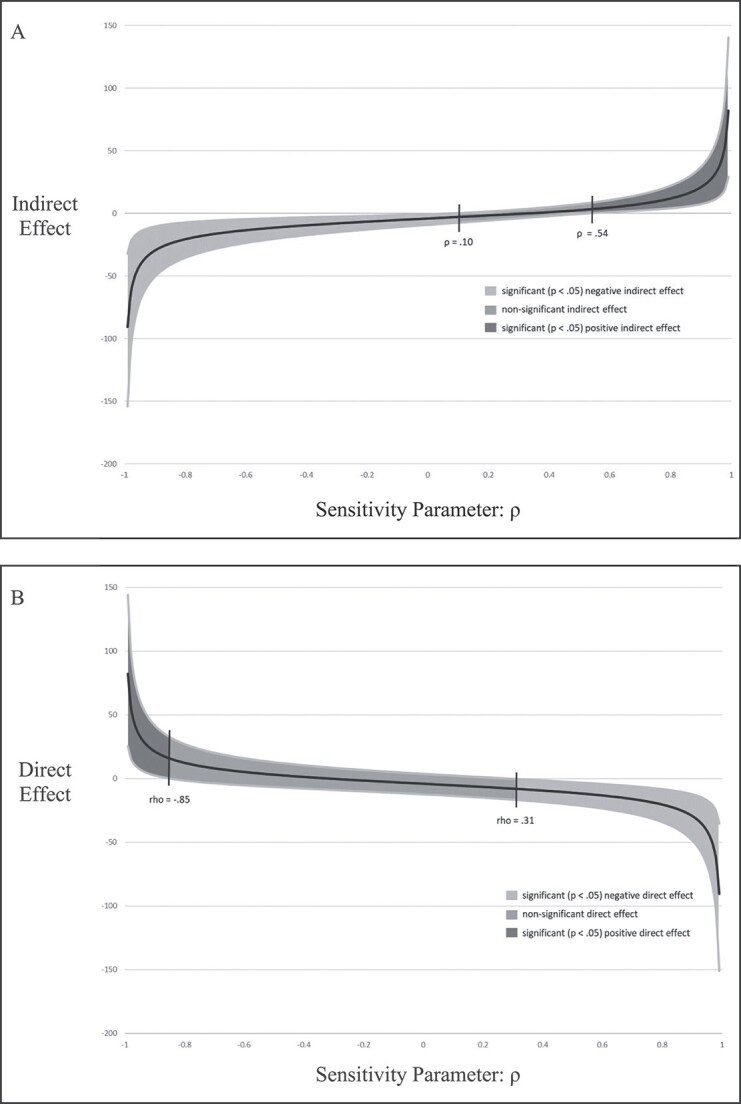
(A and B) Graphical representation of sensitivity analyses results examining differing values of the mediator (FoS) and outcome (PTSD) residual correlation (*ρ*) effects on the indirect effect (A) and direct effect (B) . The shaded portion represents the 95% credibility interval. From *ρ* = −.99 to .10, the indirect effect is negative and significant; from *ρ* = .11 to .54, the indirect effect is not significant; from .55 to .99, the indirect effect is positive and significant. The shaded portion represents the 95% credibility interval. From *ρ* = −.99 to −.85, the direct effect is positive and significant; from *ρ* = −.84 to .30, the direct effect is not significant; from .31 to .99, the direct effect is negative and significant.

To account for unmeasured confounders, sensitivity analyses fixed the residual correlation among the mediator (FoS) and outcome (PTSD) at various levels (−.99 to .99). The indirect effect results suggest that the indirect effect remains statistically significant from −.99 up to .09 (indirect effect_(*ρ* = −.99)_ = −90.57; indirect effect_(*ρ* = .09)_ = −3.00; see Figure 2, A). From *ρ* = .10 to .53, the indirect effect becomes statistically not significant, and from *ρ* = .54 to .99 the indirect effect is positive and statistically significant (indirect effect_(*ρ* = .54)_ = 3.35; indirect effect_(*ρ* = .99)_ = 81.82). Thus, while some could argue that the indirect effect is not particularly robust, direct effects become negative and significant at a *ρ*-value of .31 and remain significant through *ρ* = .99 (direct effect = −8.12_(*ρ* = .31)_; −90.5_(*ρ* = .99)_; see Figure 2, B), with beneficial effects of CBTi on PTSD becoming statistically nonsignificant in the narrow range of residual correlations between FoS and PTSD of .10 to .31.

## Discussion

Our results demonstrated that following a version of CBTi adapted to address FoS there was a significant decrease in FoS scores. Post-CBTi FoSI scores were positively related to later PTSD symptoms with reductions in FoS mediating the relationship between CBTi and PTSD symptoms following the subsequent CPT intervention. Changes in FoS following CBTi may partly explain how CBTi boosted the effects of CPT for PTSD severity.

The main finding is consistent with Werner et al.’s model that proposes FoS leads to maladaptive thoughts and behaviors that contribute to the development and maintenance of insomnia [9]. Our CBTi approach was tailored to address any such maladaptations. Given that the post-CBTi reductions in FoS were significantly larger than those in the control condition, this does suggest a direct effect of adapted CBTi on FoS. The results included a raw reduction of 17.3 points in mean (SD) FoSI scores following adapted CBTi [from 40.3(19.5) to 22.9(20.4)] representing a large within-group effect (Hedges *g* = −0.85). This compares to the 14.1 point reduction [from 28.4(15.5) to 14.3(15.3); *g* = −0.89] in the Kanady study, which did not adapt CBTi to address FoS [15] and had a smaller, less clinically severe sample (see Table 1). Although it is therefore difficult to ascertain whether the addition of FoS-focused strategies to standard CBTi contributes to any greater reductions in FoS than standard CBTi alone, we offer some clinical considerations subsequently.

**Table 1:** Comparison of baseline characteristics of participants in the CBTi condition of the current study and the Kanady et al. study

	Current study	Kanady et al. [15, 42]
CBTi sample size, *N*	56	29
Age, mean (SD), years	34.2 (10.8)	37.1 (10.4)
Female (%)	96.4	75.9
Current depression (%)	96.4	17.2
Psychotropic medication use (%)	23.2	37.9
Current/prior PTSD treatment (%)	37.5	100
PTSD duration, mean (SD), years	8.2 (12.1)	20.4 (13.6)
ISI, mean (SD)	20.9 (3.8)	19.7 (3.6)
CAPS, mean (SD)^a^	63.4 (14.7)	51.5 (18.2)
FoSI score, mean (SD)	40.3 (19.5)	28.1 (15.5)

^a^CAPS score excludes insomnia, nightmare, and hypervigilance items. Abbreviations: CAPS, Clinician Administered PTSD Scale; CBTi, cognitive behavioral therapy for insomnia; ISI, Insomnia Severity Index.

The study limitations include a sample comprising predominately women with IPV, limiting generalizability to other trauma populations, especially when considering that gender and trauma type may influence PTSD symptomology [42, 43]. In addition, the shared variance between FoS and PTSD suggests unmeasured variables account for variance in both post-CBTi FoS and post-CPT PTSD. Some of these omitted variables are likely shared between both FoS and PTSD. Although this shared variance is concerning, sensitivity analyses were employed to account for unmeasured confounders. Furthermore, it is possible that an unaccounted-for variable contributes to changes in both FoS and PTSD. For example, CBTi may decrease the frequency of trauma-related nightmares. This has previously been demonstrated [44], but it has also been shown that nightmares were not reduced by our adapted CBTi [45]. Finally, the current study was not designed to directly compare versions of CBTi that do, versus do not, incorporate adaptations that target FoS.

Nonetheless, our adaptations to CBTi to address FoS were feasible to implement and deliver. The approach is consistent with a case-conceptualization approach to CBTi delivery that embraces such adaptations [46]. Further, there is relatively low patient and clinician burden in administering the FoSI and addressing the highly rated items during CBTi. In the current study, we used the original 23-item version of the FoSI, but a 13-item short form (FoSI-SF) has been validated [12] providing an even lower burden option. Until further guidance is available, we suggest as a practical approach to use the FoSI-SF to identify items that participants rate high (>3 on the 0–4 scale) and to address them if determined with the participant that these items are contributing to disrupted sleep.

For at least two decades the PTSD field has advocated for identifying and targeting variables that may improve the effectiveness of PTSD treatments [47]. As demonstrated in the parent study [18], insomnia is one such variable that is modifiable. The current findings demonstrate that FoS may also be reduced by CBTi among individuals with severe PTSD and co-occurring depression and those reductions may have meaningful effects on subsequent trauma-focused outcomes. Future research is needed to ascertain whether FoS adaptations provide additional benefits to CBTi across various subsamples of individuals with PTSD as well as those exhibiting FoS in the absence of PTSD. Identifying those patient characteristics that support the inclusion of FoS focused adaptations to treatment will be especially important to guide clinical practice.
